# Supplementary material for: Registered Drug Packs of Antimicrobials and Treatment Guidelines for Prostatitis: Are They in Accordance?
Source: Healthcare (Basel). 2022 Jun 22;10(7):1158. doi: 10.3390/healthcare10071158 (PMC9322485; doi:10.3390/healthcare10071158)

Table S1. Comparison of prostatitis treatment guidelines

ISKRA [1]		NICE [2]		AFP [3]		ATGAR [4]		SSID [5]	IIUS [6]	
Acute bacterial prostatitis										
First choice	3rd generation cephalosporins ± aminoglycosides<br>or<br>Aminopenicillins + beta-lactamase inhibitors<br>or<br>Ureidopenicillins + beta-lactamase inhibitors<br>or<br>Fluoroquinolones (ciprofloxacin, levofloxacin),<br>Parenteral 7 – 10 days, followed by oral fluoroquinolones for further 2 – 4 weeks	First-choice oral antibiotics (guided by susceptibilities when available)	Ciprofloxacin oral 2 x 500 mg, 2 weeks Ofloxacin 2 x 200 mg, 2 weeks	Mild or moderate disease while awaiting culture	Trimethoprim oral 1 x 300 mg 2 weeks, or<br>Cephalexin oral 2 x 500 mg, 2 weeks<br>or<br>Amoxicillin and clavulanic acid 2 x 500 mg + 125 mg oral, 2 weeks	First choice	Ciprofloxacin oral 2 x 500 mg, 4 weeks<br>Levofloxacin oral 1 x 500 mg, 4 weeks<br>Cefixime oral 1 x 400 mg, 4 weeks<br>These antibiotics can be combined with an aminoglycoside	Ciprofloxacin 400 mg/12 h i. v. or oral 2 x 500 mg, 2 – 4 weeks<br>Levofloxacin 500 mg/24 h i. v. a or oral 1 x 500 mg, 2 – 4 weeks<br>Trimethoprim-sulfamethoxazole oral 2 x 160/800 mg, 2 – 4 weeks<br>Gentamicin 5 mg/kg a day with or without ampicilin 2 g/6 h i. v.	Empirical treatment	Levofloxacin oral 1-2 x 500 mg, 2 – 4 weeks<br>Trimethoprim-sulfamethoxazole oral 2 x 160/800 mg, 2 – 4 weeks
		Alternative first-choice oral antibiotic if a fluoroquinolone antibiotic is not appropriate (seek specialist advice; guided by susceptibilities when available)	Trimethoprim oral 2 x 200 mg, 2 weeks			Alternative	Trimethoprim-sulfamethoxazole oral 2 x 160/800 mg,<br>4 weeks		if allergic to a fluoroquinolone	Amoxicillin and clavulanic acid oral 3 x 1 g, 2 – 4 weeks
		First-choice intravenous antibiotics (if unable to take oral antibiotics or severely unwell; guided by susceptibilities when available).<br>Antibiotics may be combined if sepsis a concern	Ciprofloxacin 400 mg twice or three times a day Levofloxacin 500 mg once a day Cefuroxime: 1.5 g three or four times a day Ceftriaxone: 2 g once a day Gentamicin: Initially 5 mg/kg to 7 mg/kg once a day, subsequent doses adjusted according to serum gentamicin concentration. Amikacin: Initially 15 mg/kg once a day (maximum per dose 1.5 g once a day), subsequent doses adjusted according to serum amikacin concentration (maximum 15 g per course).	Appears septic or unable to tolerate oral therapy	Admit to hospital, offer parenteral therapy with ampicillin and gentamycin or ceftriaxone as per severe pyelonephritis treatment	Appears septic	Hospital referral for initial intravenous treatment		Appears septic	Ceftriaxone i.v. 1 x 2 g<br>Gentamicin i.v. 3 mg/kg<br>Levofloxacin i.v.1 x 500 mg
Second choice	Carbapenems, Parenteral 7 – 10	Second-choice oral antibiotics	Levofloxacin oral 1 x 500 mg, 2 weeks then review							

	days, followed by oral fluoroquinolones for further 2 – 4 weeks<br>or<br>Trimethoprim-sulfamethoxazole, Parenteral/Oral 2 – 3 weeks	(after discussion with specialist)	Trimethoprim-sulfamethoxazole<br>oral 2 x 160/800 mg, 2 weeks then review Trimethoprim-sulfamethoxazole<br>should only be considered when there is bacteriological evidence of sensitivity and good reasons to prefer this combination to a single antibiotic							
		Second-choice intravenous antibiotics	Consult a local microbiologist							
Chronic bacterial prostatitis										
First choice	Fluoroquinolones (ciprofloxacin, levofloxacin, ofloxacin)<br>or<br>Trimethoprim-sulfamethoxazole, Oral, 4 – 6 weeks			First choice	Norfloxacin oral 2 x 400 mg, 4 weeks, or Trimethoprim oral 1 x 300, 4 weeks	First choice	Ciprofloxacin oral 2 x 500 mg, 4-6<br>Weeks<br>or<br>Levofloxacin oral 1 x 500 mg, 4 weeks	Ciprofloxacin oral 2 x 500 mg, 6 – 12 weeks<br>Levofloxacin oral 1 x 500 mg, 6 – 12 weeks<br>Trimethoprim-sulfamethoxazole oral 2 x 960 mg, 4 – 6 weeks		Levofloxacin oral 1-2 x 500 mg, 4 – 6 weeks<br>Ciprofloxacin oral 2 x 750 mg, 4 – 6 weeks
Alternative	Macrolides, Oral, 4 – 6 weeks.<br>Possible and useful administration in combination with fluoroquinolones azithromycin 500 mg daily,<br>only for the first three days of each treatment week<br>or<br>3rd generation oral cephalosporins ± parenteral aminoglycosides, 7 – 10 days then switch to oral			If chlamydia or ureaplasma noted	Doxycycline oral 2 x 100 mg 2–4 weeks	Alternative	Trimethoprim sulfamethoxazole Oral 2 x 160/800 mg,<br>4 weeks		If chlamydia or micoplasma noted	Azithromycin oral 1 x 500 mg<br>Doxycycline oral 2 x 100 mg 2 weeks

	fluoroquinolones or trimethoprim-sulfamethoxazole for 2 – 4 weeks									

The numbers in the brackets represent literature.

Table S2. AGREE analysis of the included guidelines (available from: https://www.agreetrust.org/resource-centre/agree-reporting-checklist/)

	ISKRA [1]	NICE [2]	AFP [3]	ATGAR [4]	SSID [5]	IUS [6]
DOMAIN 1: SCOPE AND PURPOSE	79.6%	90.7%	42.5%	83.3%	40.7%	53.7%
DOMAIN 2: STAKEHOLDER INVOLVEMENT	72.2%	83.3%	38.8%	79.6%	42.5%	51.8%
DOMAIN 3: RIGOUR OF DEVELOPMENT	70.8%	86.8%	19.4%	71.5%	17.3%	63.5%
DOMAIN 4: CLARITY OF PRESENTATION	81.0%	92.5%	74.1%	64.8%	55.5%	53.7%
DOMAIN 5: APPLICABILITY	34.7%	65.2%	19.4%	36.1%	22.2%	27.7%
DOMAIN 6: EDITORIAL INDEPENDENCE	44.4%	30.5%	5.5%	38.8%	2.7%	2.7%

The numbers in the brackets represent literature.

Table S3. Accordance of oral antibiotics in the ISKRA[1] treatment guidelines for prostatitis with drug packs available in selected countries [1].

ISKRA[1] recommendation [1]	Summary of recommendation	Croatia [7]	United Kingdom [8]	Australia [9]	Slovenia [10]	Italy [11]	Spain [12]
Treatment of acute bacterial prostatitis							
Oral Fluoroquinolones, 2 – 4 weeks after parenteral treatment	Ciprofloxacin 2 x 500 – 750 mg 2 – 4 weeks	3 – 6 packs of 10 500 mg ± 3 – 6 packs of 10 250 mg, excess **2 or 4 units** (1 or 2 days); single dose 750 mg not registered	1 – 2 packs of 28 500 mg units or 2 – 4 packs of 14 750 mg units, **matched**	1 – 2 packs of 28 500 or 750 mg units, **matched**	3 – 6 packs of 10 units 500 or 750 mg, excess **2 or 4 units** (1 or 2 days)	1 – 2 packs of 28 units 500 or 750 mg, **matched**	2 – 4 packs of 14 units of 500 or 750 mg, **matched**
	Levofloxacin 1 x 500 mg 2 – 4 weeks	1 – 2 packs of 14 units, **matched**	2 – 4 packs of 7 units, **matched**	Only bulk, not applicable	2 – 3 packs of 10 units, excess **6 or 2 units** (6 or 2 days)	1 pack of 14 or 28 units, **matched**	1 – 2 packs of 14 units, **matched**
	Norfloxacin 2 x 400 mg 2 – 4 weeks	2 – 3 packs of 20 units, excess **12 or 4 units** (6 or 2 days)	Not registered	1 – 2 packs of 14 units, **matched**	2 – 3 packs of 20 units, excess **12 or 4 units** (6 or 2 days)	2 – 4 packs of 14 units, **matched**	2 – 4 packs of 14 units, **matched**
Trimethoprim / sulfamethoxazole, oral 2 – 3 weeks	Trimethoprim / sulfamethoxazole 2 x 160/800 mg 2 – 3 weeks	2 – 3 packs of 20 units, excess **12 or 18 units** (6 or 9 days)	2 – 3 packs of 14 units, **matched**	3 – 5 packs of 10, excess **2 or 8 units** (1 or 4 days)	3 – 5 packs of 20 80/400 mg units, excess **4 or 16 half dose units** (1 or 4 days)	2 – 3 packs of 16 units, excess **4 or 6 units** (2 or 3 days)	2 – 3 packs of 20 units, excess **12 or 18 units** (6 or 9 days)
Treatment of chronic bacterial prostatitis							
Fluoroquinolones (ciprofloxacin, levofloxacin, ofloxacin), oral 4 – 6 weeks	Ciprofloxacin 2 x 500 – 750 mg 4 – 6 weeks	6 – 9 packs of 10 500 mg ± 6 – 9 packs of 10 250 mg, excess **4 or 6 units** (2 or 3 days); single dose 750 mg not registered	2 – 3 packs of 28 500 mg units or 4 – 6 packs of 14 750 mg units, **matched**	2 – 3 packs of 28 500 or 750 mg units, **matched**	6 – 9 packs of 10 500 or 750 mg, excess **4 or 6 units** (2 or 3 days)	2 – 3 packs of 28 units 500 or 750 mg, **matched**	4 – 6 packs of 14 units of 500 or 750 mg, **matched**
	Levofloxacin 1 x 500 mg 4 – 6 weeks	2 – 3 packs of 14 units, **matched**	4 – 6 packs of 7 units, **matched**	Only bulk, not applicable	3 – 5 packs of 10 units, excess **2 or 8 units** (2 or 8 days)	1 pack of 28 units – 1 pack of 28 and 1 pack of 14 units, **matched**	2 – 3 packs of 14 units, **matched**
	Ofloxacin 2 x 200 mg 4 – 6 weeks	Not registered (only eye drops)	4 – 6 packs of 14 units, **matched**	Not registered (only eye drops)	Not marketed	Not marketed	4 – 6 packs of 14 units, **matched**
Trimethoprim / sulfamethoxazole, oral 4 – 6 weeks	Trimethoprim / sulfamethoxazole 2 x 160/800 mg 4 – 6 weeks	3 – 5 packs of 20 units, excess **4 or 16 units** (2 or 8 days)	4 – 6 packs of 14 units, **matched**	6 – 9 packs of 10, excess **4 or 6 units** (2 or 3 days)	6 – 9 packs of 20 80/400 mg units, excess **8 or 12 half dose units** (2 or 3 days)	4 – 6 packs of 16 units, excess **8 or 12 units** (4 or 6 days)	3 – 5 packs of 20 units, excess **4 or 16 units** (2 or 8 days)
Macrolides, oral 4 – 6 weeks. Possible and useful administration in combination	Azithromycin 1 x 500 mg 3 times a week, 4 – 6 weeks	4 – 6 packs of 3 units, **matched**	1 pack of 12 units – 1 pack of 12 and 1 pack of 6 units, **matched**	4 – 6 packs of 3 units, **matched**	4 – 6 packs of 3 units, **matched**	4 – 6 packs of 3 units, **matched**	4 – 6 packs of 3 units, **matched**

with fluoroquinolones, azithromycin 500 mg daily, only for the first three days of each treatment week							
3rd generation oral cephalosporins ± parenteral aminoglycosides 7 – 10 days then switch to oral fluoroquinolones or trimethoprim / sulfamethoxazole for 2 – 4 weeks	Cefixime 1 x 400 mg or 2 x 200 mg 7 – 10 days	1 pack of 10 400 mg units, excess 3 units (3 days) or **matched** if treated for 10 days	1 pack of 14 200 mg units – 10 packs of 2 200 mg units, **matched**	Not registered	1 pack of 10 400 mg units, excess 3 units (3 days) or **matched** if treated for 10 days	1 pack of 7 or 10 units, **matched**	1 pack of 10 400 mg units, excess 3 units (3 days) or **matched** if treated for 10 days
oral fluoroquinolones or trimethoprim / sulfamethoxazole for 2 – 4 weeks	Fluoroquinolones – as for Treatment of acute bacterial prostatitis above						
	Trimethoprim/sulfamethoxazole 2 x 160/800 mg 2 – 4 weeks – as above						
Treatment of prostatitis caused by *C. trachomatis*, *U. urealyticum* and *M. genitalium*							
Azithromycin in a total dose of 4.5 g orally, for 3 weeks administered as PULSED THERAPY –	Azithromycin 1 x 500 mg 3 times a week, 3 weeks	3 packs of 3 units, **matched**	3 packs of 3 units or 1 pack of 6 and 1 pack of 3 units, **matched**	3 packs of 3 units, **matched**	3 packs of 3 units, **matched**	3 packs of 3 units, **matched**	3 packs of 3 units, **matched**
Levofloxacin 500 mg orally every day for 2 – 4 weeks	Levofloxacin 1 x 500 mg 2 – 4 weeks	As Treatment of acute bacterial prostatitis					
Ofloxacin 2 × 300 mg up to 2 × 400 mg orally during 2 to 4 weeks	Ofloxacin 2 × 300 mg up to 2 × 400 mg 2 to 4 weeks	Not registered	300 mg not registered, 2 – 4 14 400 mg unit packs, **matched**	Not registered	Not marketed	Not marketed	4 – 8 14 units 200 mg packs, **matched**
Doxycycline 2 × 100 mg orally per day for 3 – 4 weeks	Doxycycline 2 × 100 mg 3 – 4 weeks	2 – 3 packs of 25 units, excess **8 or 44 units** (4 or 22 days)	3 packs of 7 units – 1 pack of 56 units, **matched**	2 packs of 21 units – 8 packs of 7 units, **matched**	6 – 7 packs of 8 units, excess **6 units** (3 days) or **matched** if treated for 4 weeks	6 – 7 packs of 8 units, excess **6 units** (3 days) or **matched** if treated for 4 weeks	2 packs of 21 units – 4 packs of 14 units, **matched**
Treatment of inflammatory chronic pelvic pain/nonbacterial prostatitis							
Empirical antimicrobial therapy (fluoroquinolones, trimethoprim / sulfamethoxazole, doxycycline) orally/2 weeks, In case of clinical improvement prolong to 4 weeks	Doxycycline 2 × 100 mg 2 – 4 weeks	2 – 3 packs of 25 units, excess **22 or 44 units** (11 or 22 days)	1 pack of 28 – 1 pack of 56 units, **matched**	4 – 8 packs of 7 units, **matched**	4 – 7 packs of 8 units, excess 4 units (2 days) or **matched** if treated for 4 weeks	1 pack of 20 and one pack of 10 units – 7 packs of 8 units, excess **2 units** (1 day) or **matched** if treated for 4 weeks	2 – 4 packs of 14 units, **matched**
	Other – as above						

[1] The Intersectoral Coordination Mechanism for the Control of Antimicrobial Resistance; The numbers in the brackets represent literature.

References:

1. Višnja Škerk GŠ, Ognjen Kraus, Jasmina Vraneš, Edita Sušić,, Suzana Bukovski TH, Željko Kaštelan, Vesna Mađarić,, Alemka Markotić SMM, Dragan Soldo,, Dalibor Vukelić ATA. ISKRA Guidelines On Diagnostics and Treatment Of Prostatitis – Croatian National Guidelines. *Liječ Vjesn* **2017**, *139*, 254-267.
2. National Institute for Health and Care Excellence guideline N. Prostatitis (acute): antimicrobial prescribing (NG110). 2018. Available from: https://www.nice.org.uk/guidance/ng110 [Accessed: 28th January 2022].
3. Dickson G. Prostatitis Diagnosis and treatment. *Australian Family Physician* **2013**, *42*, 4.
4. Fernández Urrusuno R, Serrano Martino C. *Guía Terapéutica Antimicrobiana del Área Aljarafe,* 3ª ed.; online, 2018; pp. 159. Available from: https://portal.guiasalud.es/wp-content/uploads/2018/12/GPC_578_Antimicrobianos_Aljarafe_2018.pdf [Accessed: 28th January 2022]
5. Logar, M. Urogenital Tract Infections. *Farm Vestn* **2018**, *69*, 114-121.
6. Società Italiana di Urologia. *Raccomandazioni in tema di Infezioni delle vie urinarie*, 1st ed.; online, 2015.pp. 149. Available from: https://www.siu.it/linee-guida/raccomandazioni-siu/2015-raccomandazioni-siu/raccomandazioni-in-tema-di-infezioni-delle-vie-urinarie [Accessed: 28th January 2022]
7. Agency for Medicinal Products and Medical Devices. Medicinal Products Database. Available from: http://www.halmed.hr/en/Lijekovi/Baza-lijekova/ [Accessed: 28th January 2022].
8. The electronic medicines compendium. Available from: https://www.medicines.org.uk/emc#gref [Accessed: 28th January 2022].
9. The Therapeutic Goods Administration. ARTG Search. Available from: https://tga-search.clients.funnelback.com/s/search.html?query=&collection=tga-artg [Accessed: 28th January 2022].
10. MZ JAZMP ZZZS NIJZ. Centralna Baza Zdravil. Available from: http://www.cbz.si/cbz/bazazdr2.nsf/Search/$searchForm?SearchView [Accessed: 28th January 2022].
11. Agenzia Italiana del Farmaco. La Banca Dati Farmaci. Available from: https://farmaci.agenziafarmaco.gov.it/bancadatifarmaci/home [Accessed: 28th January 2022].
12. CIMA. Find your medicine here. Available from: http://cima.aemps.es/cima/publico/home.html [Accessed: 28th January 2022].

Figure S1. Flowchart of selection of guidelines

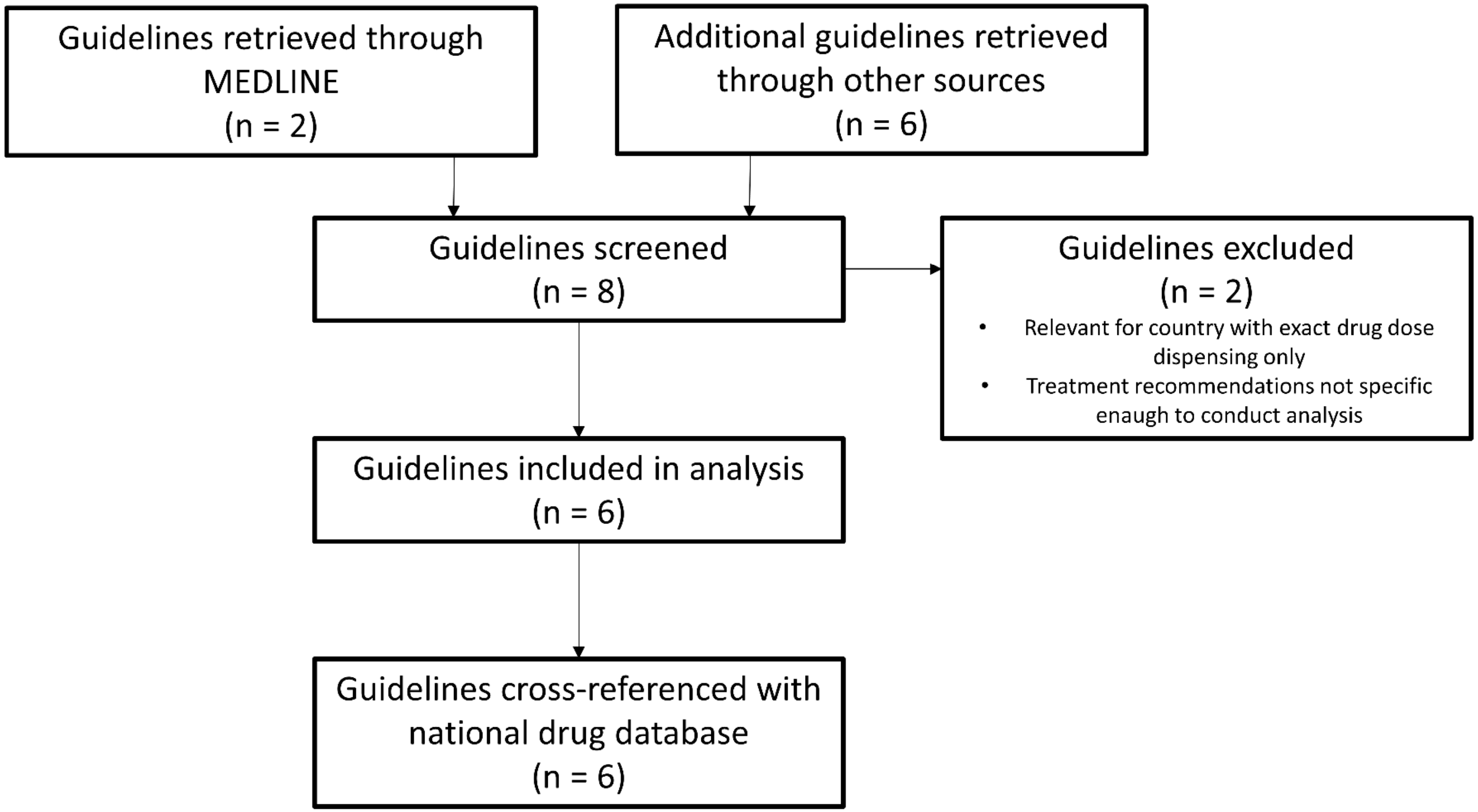

Supplement: Supplementary file 1 [file healthcare-10-01158-s001.zip › healthcare-1742593-supplementary.pdf]
